# The Temporal Pattern of Changes in Serum Biomarker Levels Reveals Complex and Dynamically Changing Pathologies after Exposure to a Single Low-Intensity Blast in Mice

**DOI:** 10.3389/fneur.2015.00114

**Published:** 2015-06-12

**Authors:** Farid Ahmed, Stefan Plantman, Ibolja Cernak, Denes V. Agoston

**Affiliations:** ^1^Department of Anatomy, Physiology and Genetics, Uniformed Services University, Bethesda, MD, USA; ^2^Department of Neuroscience, Karolinska Institutet, Stockholm, Sweden; ^3^Faculty of Rehabilitation Medicine, Canadian Military and Veterans’ Clinical Rehabilitation Research, University of Alberta, Edmonton, AB, Canada

**Keywords:** blast, traumatic brain injury, mouse, serum, biomarkers

## Abstract

Time-dependent changes in blood-based protein biomarkers can help identify the ­pathological processes in blast-induced traumatic brain injury (bTBI), assess injury severity, and monitor disease progression. We obtained blood from control and injured mice (exposed to a single, low-intensity blast) at 2-h, 1-day, 1–week, and 1-month post-injury. We then determined the serum levels of biomarkers related to metabolism (4-HNE, HIF-1α, ceruloplasmin), vascular function (AQP1, AQP4, VEGF, vWF, Flk-1), inflammation (OPN, CINC1, fibrinogen, MIP-1a, OX-44, p38, MMP-8, MCP-1 CCR5, CRP, galectin-1), cell adhesion and the extracellular matrix (integrin α6, TIMP1, TIMP4, Ncad, connexin-43), and axonal (NF-H, Tau), neuronal (NSE, CK-BB) and glial damage (GFAP, S100β, MBP) at various post-injury time points. Our findings indicate that the exposure to a single, low-intensity blast results in metabolic and vascular changes, altered cell adhesion, and axonal and neuronal injury in the mouse model of bTBI. Interestingly, serum levels of several inflammatory and astroglial markers were either unchanged or elevated only during the acute and subacute phases of injury. Conversely, serum levels of the majority of biomarkers related to metabolic and vascular functions, cell adhesion, as well as neuronal and axonal damage remained elevated at the termination of the experiment (1 month), indicating long-term systemic and cerebral alterations due to blast. Our findings show that the exposure to a single, low-intensity blast induces complex pathological processes with distinct temporal profiles. Hence, monitoring serum biomarker levels at various post-injury time points may provide enhanced diagnostics in blast-related neurological and multi-system deficits.

## Introduction

The mild form of traumatic brain injury (mTBI, also called concussion) accounts for ~75% of all head injuries among civilians and military personnel (1, 2). In the U.S. military, mTBI, mostly caused by explosive weaponry, has affected ~300,000–500,000 military personnel during the last 15 years (3–5). Numerous terms, including blast-induced neurotrauma (BINT) (3, 4), blast-induced TBI, or just blast TBI (bTBI), have been coined to describe the unique clinical entity of blast-induced neurological deficits caused by interwoven mechanisms of systemic, local, and cerebral responses to blast exposure(s) (4, 5).

Similar to civilian concussion, the acute symptoms of mild, blast-induced TBI (mbTBI) are usually mild and transient (6). However, in addition to the large number of affected individuals, mbTBI is a significant health issue for several reasons. Repetitive mild injuries – if they occur within the period of increased cerebral vulnerability – can result in a disproportionally severe acute outcome (7, 8). Moreover, mbTBI, especially when repeated, could significantly increase the probability of late onset neurodegenerative conditions, such as chronic traumatic encephalopathy (CTE) (9, 10). Finally, blast interacting with the entire body and not only with the head, induces complex co-occurring systemic, local, and cerebral responses to injury (11–14). Thus, the neurological deficits that develop after blast exposure(s) are resulted not only from the brain damage caused via head-blast interaction but also from multiple, synergistically acting injury mechanisms. Because of such complexity, the diagnosis of blast-induced neurological consequences, both during the acute and chronic injury phases, is extremely challenging: the manifestations of mbTBI are often masked by symptoms of systemic changes.

It is noteworthy that many of the acute and subacute neurobehavioral symptoms of mbTBI, such as impaired cognition and emotional instability, are shared with post-traumatic stress disorder (PTSD), a complex clinical syndrome triggered by extreme psychological stress without physical injury (15). Since the diagnosis of mbTBI and PTSD are often solely based on neuropsychological tests and self-reported information, the differential diagnosis is challenging due to the absence of objective outcome measures. While earlier studies using rodent models have suggested that the pathobiology of PTSD and mbTBI may involve distinct mechanisms (15–18), the precise identities of the molecular events leading to the observed functional changes are still unclear (19).

The increasing numbers of research studies using various animal models, in combination with analytical techniques such as histopathology and proteomics, have identified some of the mechanisms underlying the pathology of bTBI. These findings include metabolic and vascular changes; axonal, neuronal and glial damage; inflammation; and altered cell adhesion, among others (13, 20–29). In this study, we used the previously developed, well-standardized, and validated mouse model of primary blast injuries (30), which reproduces the main consequences of blast exposures: changes in general physiology, multi-organ damages, systemic compensatory mechanisms, as well as neurological deficits seen in individuals exposed to differing blast intensity levels. Our intention was to reproduce the full spectrum of injury response mechanisms induced by primary blast and not only head trauma as an isolated entity (30, 31). Previous works with this model has determined that the vital functions, memory and cognitive performance, and behavioral impairments are comparable with the symptoms of mild and moderate TBI in war fighters exposed to blast (30, 31). Those findings also implicated inflammation as a potential mechanistic explanation for the long-term neurological deficits after blast exposure.

Using the same mouse model of bTBI (30, 31), we determined changes in the serum levels of protein biomarkers at 2 h, 1 day, 1 week, and 1 month after exposure. We selected 31 biomarkers indicative of changes in metabolic and vascular functions; cell adhesion; axonal, neuronal, and glial integrities; and inflammation to determine the temporal pattern of changes from early/acute (2 h) to late/chronic (1 month) post-injury time points. We found that the exposure to low-intensity blast triggers complex changes in serum levels of biomarkers, many of them remaining highly elevated at 1 month after the injury suggesting that a single exposure to low-intensity blast can induce lasting molecular pathologies. Our findings have implications for determining the period of ICV after mbTBI, and identifying the distinct pathologies that involve a widespread systemic response in the pathobiology of blast-induced neurological deficits. Moreover, our results underline the role of long-term monitoring of serum protein biomarkers in the differential diagnosis between mbTBI and PTSD as well as mbTBI and impact TBI. As such, our findings imply that continuous assessments of serum protein biomarkers could help in predicting the outcome of mbTBI and preempting multi-organ complications of blast exposure.

## Materials and Methods

All protocols involving the use of animals complied with the Guide and Care and Use of laboratory Animals published by the NIH (DHEW publication NIH 85-23-2985) and were approved by the Johns Hopkins University Animal Use Committee. Exposures were performed at the Johns Hopkins University Applied Physics Laboratory (JHU/APL) using a modular, multi-chamber shock tube capable of reproducing complex shock wave signatures seen in theater (30, 31).

### Blast injury

Twenty-five 3–4-month-old C57/B16 mice (Jackson Laboratories) were used. Animals (weighing 22–24 g at the beginning of the study) were divided into three experimental groups: naïve (*n* = 5), sham (*n* = 5), and injured (*n* = 20). Animals were housed in pairs in standard mouse cages in a reverse 12 h light and 12 h dark cycle with access to food and water *ad libitum*. Animals in the sham and in the injured groups were anesthetized with 4% isoflurane evaporated in a gas mixture containing 30% oxygen/70% nitrous oxide and applied through a nose mask. The animals were allowed to breathe spontaneously without tracheal intubation. Mice were then mounted in a supine position to the animal holder and placed in the shock tube as described (30). The animal holder positioned the specimen at 53 cm (20.87˝) upstream from the driven section opening, so that only a well-formed incident shock wave loaded the animal and potential rarefactions from the tube opening were minimized. The neck, head, torso, and abdomen of the animal were fixed to the animal holder to avoid any movement, thus eliminating tertiary blast effects. The loading conditions (mean ± SD) were the following: measured membrane rupture pressure 19.2 ± 2.7 psig (132.38 ± 18.62 kPag); measured static pressure 7.5 ± 1.1 psig (51.71 ± 7.58 kPag); time pressure rise 0.010284 ± 0.000964 s; time pressure fall 0.0048 ± 0.0027; and pulse width 15.3 ± 2.9 ms. Sham animals were handled identically except exposure to the pressure wave.

### Body weight

Measurements were performed at baseline, and at 1-day, 3-day, 5-day, 1-week, and 1-month post-injury.

### Acute neurological outcome

Neurological outcome was evaluated immediately after exposure by observing the spontaneous behavior of the animal and by assessing the tail pinch reflex (time of response to pain stimulus), corneal reflex (time of response to corneal touch), and righting reflex (time of self-correction in an animal placed on its back to return to its normal upright position).

### Blast injury severity

The mortality rate was assessed at 24 h post-trauma. Injured mice were sacrificed at 2-h (*n* = 5), 1-day (*n* = 5), 1-week (*n* = 5), and 1-month (*n* = 5) post-injury. Blast injury severity in animals with lethal outcome and in survival animals at the end of the 30-day post-exposure period was performed using the Pathology Scoring System (PSS) for Blast Injuries (32) modified as previously described (12, 30, 33).

### Biosamples

Animals were anesthetized with 4% isoflurane evaporated in a gas mixture containing 30% oxygen/70% nitrous oxide and applied through a nose mask. After a midline thoracic incision, the heart was carefully exposed and blood samples were obtained via cardiac puncture and collected into BD Vacutainer SST plastic serum tube with clot activator and gel for serum separation (Ref. 367988). The blood was allowed to clot at room temperature for 40 min followed by centrifugation at 2000 *g* for 15 min. Collected supernatants were then divided into 200 μl aliquots, flash-frozen, and stored at −80°C until use.

### Sample preparation for reverse phase protein microarray assay

Serum samples were manually diluted 1:10 with 3× print buffer; samples were further diluted using a JANUS Varispan Integrator and Expanded Platform Workstation (PerkinElmer, Waltham, MA, USA) resulting in a 11-point serial dilution in triplicates in 384-well microarray plates (X7022; Fisher Scientific, Pittsburgh, PA, USA) (14, 18, 23, 34). The plates were then transferred into an Aushon 2470 Arrayer (Aushon Biosystems, Billerica, MA, USA) and printed on ONCYTE Nova single-pad nitrocellulose coated glass slides (Grace Bio-Labs, Bend, OR, USA). The Aushon 2470 Arrayer was set up with 16 pins and programed for single deposit. The spot diameter was set to 250 nm with a spacing of 500 nm between dots on the *x*-axis and 375 nm on the *y*-axis. Wash time was set at 2 s without delays. Following desiccation at 4°C overnight, slides were blocked with 5% non-fat dry milk in 1× TBS with 0.1% Tween 20 (TBST). Primary antibodies (see Table S1 in Supplementary Material) were diluted to 10× the optimal Western analysis concentration in antibody incubation buffer [0.1% BSA, protease inhibitors (EDTA-free Halt protease and phosphatase inhibitor cocktail, Thermo Fisher, Waltham, MA, USA), 1× TBS, 0.5% Tween 20] (14, 18, 23, 34). Slides were incubated with the primary antibody solution overnight at 4°C covered by a cover slip (Nunc* mSeries LifterSlips, Fisher Scientific, Pittsburg, PA, USA). The following day slides were thoroughly washed three times with TBST (1× TBS in 0.1% Tween 20) and incubated with the secondary antibodies [Alexa Fluor^®^ 635 goat anti-mouse (A-31574), Alexa Fluor^®^ 647 goat anti-rabbit (A-21245), 647 rabbit anti-goat (A-21446) or Alexa Fluor^®^ 633 donkey anti-sheep IgG (H + L) (A-21100)] (all from Invitrogen) diluted at 1:6000 in antibody incubation buffer for 1 h at room temperature. After three thorough washes in TBST followed by a single wash with 1× TBS slides were air dried and the intensity of fluorescent signals were determined in a Scan Array Express HT microarray scanner (Perkin Elmer, Waltham, MA, USA) using a 633 nm wavelength laser and a 647 nm filter.

### Data analysis

Data were imported into a Microsoft Excel-based bioinformatics program for analysis (14, 18, 23, 34). The program imports intensity data from the scanner and calculates total net intensity after local background subtraction (i.e., the intensities of secondary antibody alone and for each corresponding primary antibody) for each spot. Intensity data from the dilution series of each sample were then plotted against dilution on a log–log graph. Primary data were manually curated by linear regression of the log–log data and were plotted after the removal of flagged data. Flagged data include spot intensities in the saturation range or noise range, signal to noise ratios <2, or high variability between duplicate spots (>10–15%). The total amount of antigen is determined by the *y*-axis intercept or *y*-cept (i.e., by extrapolating the regression line to zero); reported values are log 10.

**Figure 1:**
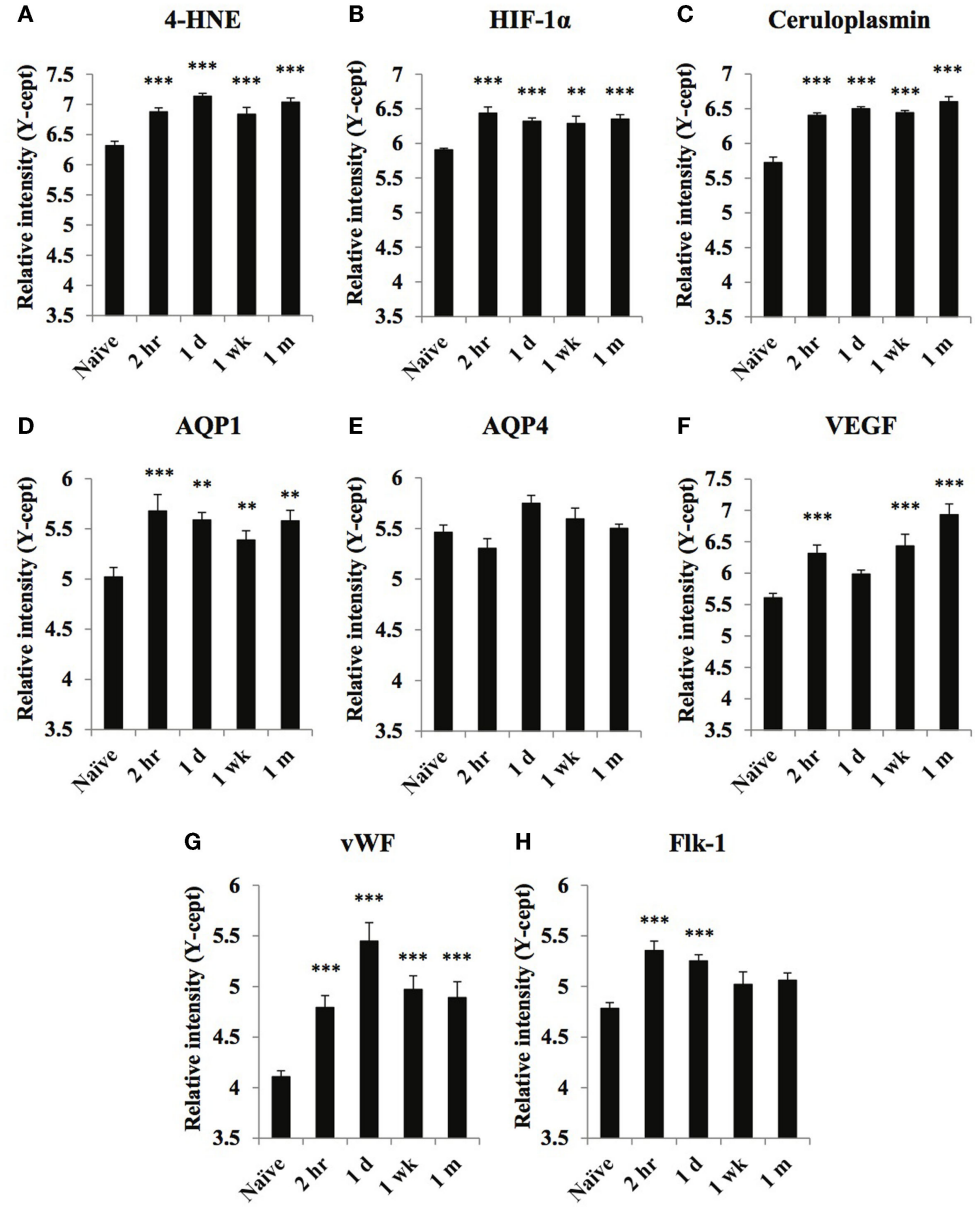
**Temporal changes of select oxidative stress and vascular markers in serum obtained from naïve and at 2 h, 1 day, 1 week, and 1 month of post-blast over pressure exposed animals**. Protein levels of oxidative stress markers 4-HNE **(A)**, HIF-1α **(B)**, ceruloplasmin **(C)**, vascular related biomarkers AQP1 **(D)**, AQP4 **(E)**, VEGF **(F)**, vWF **(G)**, and FLK-1 **(H)** were assayed using reverse phase protein microarray (RPPM) and are expressed as *y*-cept values (log 10). Data are presented as the mean ± SEM; **p < 0.01, ***p < 0.001.

**Figure 2:**
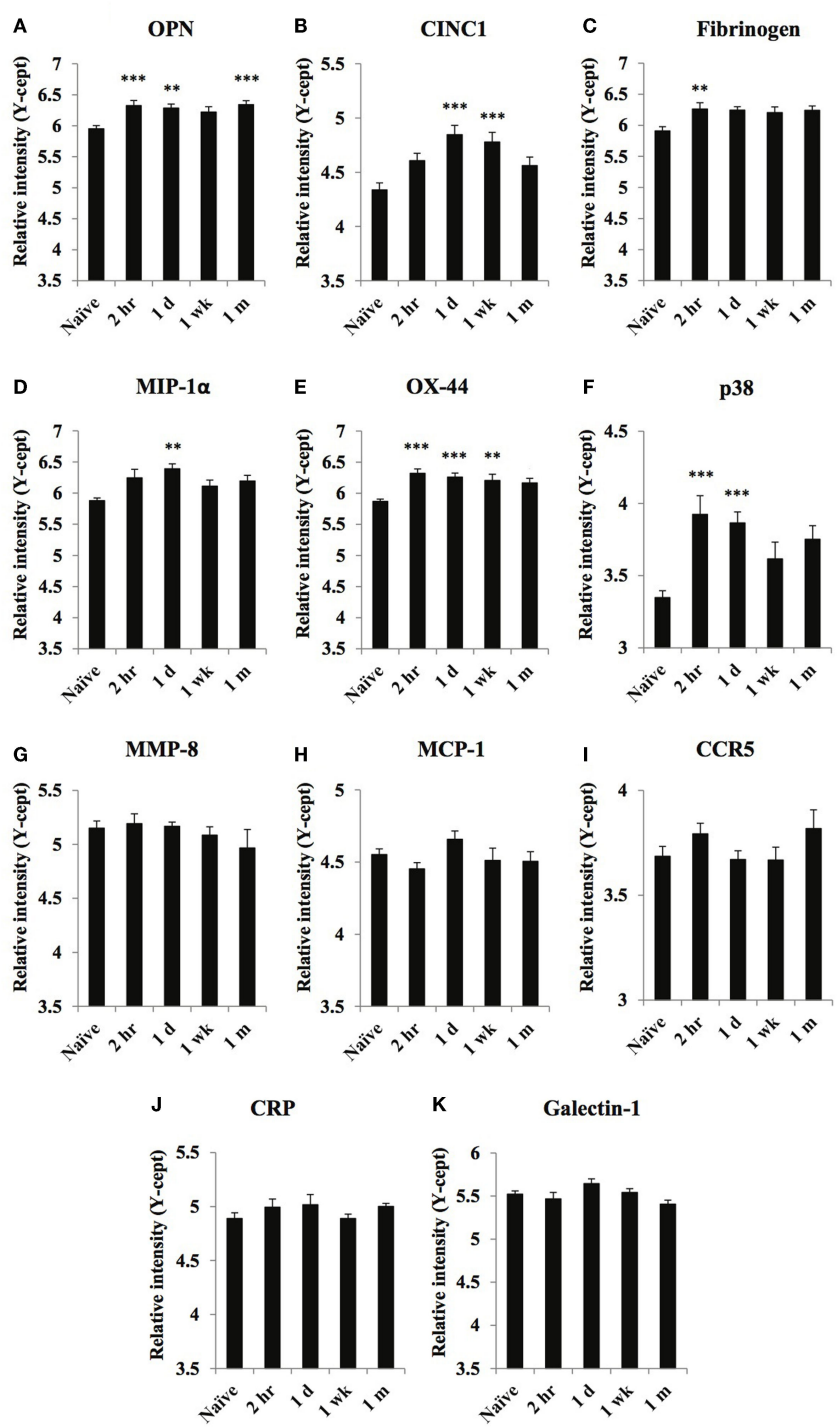
**Temporal changes of select inflammatory markers in serum obtained from naïve and at 2 h, 1 day, 1 week, and 1 month of post-blast over pressure exposed animals**. Protein levels of OPN **(A)**, CINC1 **(B)**, fibrinogen **(C)**, MIP-1α **(D)**, OX-44 **(E)**, p38 **(F)**, MMP-8 **(G)**, MCP-1 **(H)**, CCR5 **(I)**, CRP **(J)**, and galectin-1 **(K)** were assayed using reverse phase protein microarray (RPPM) and are expressed as *y*-cept values (log 10). Data are presented as the mean ± SEM; **p < 0.01, ***p < 0.001.

**Figure 3:**
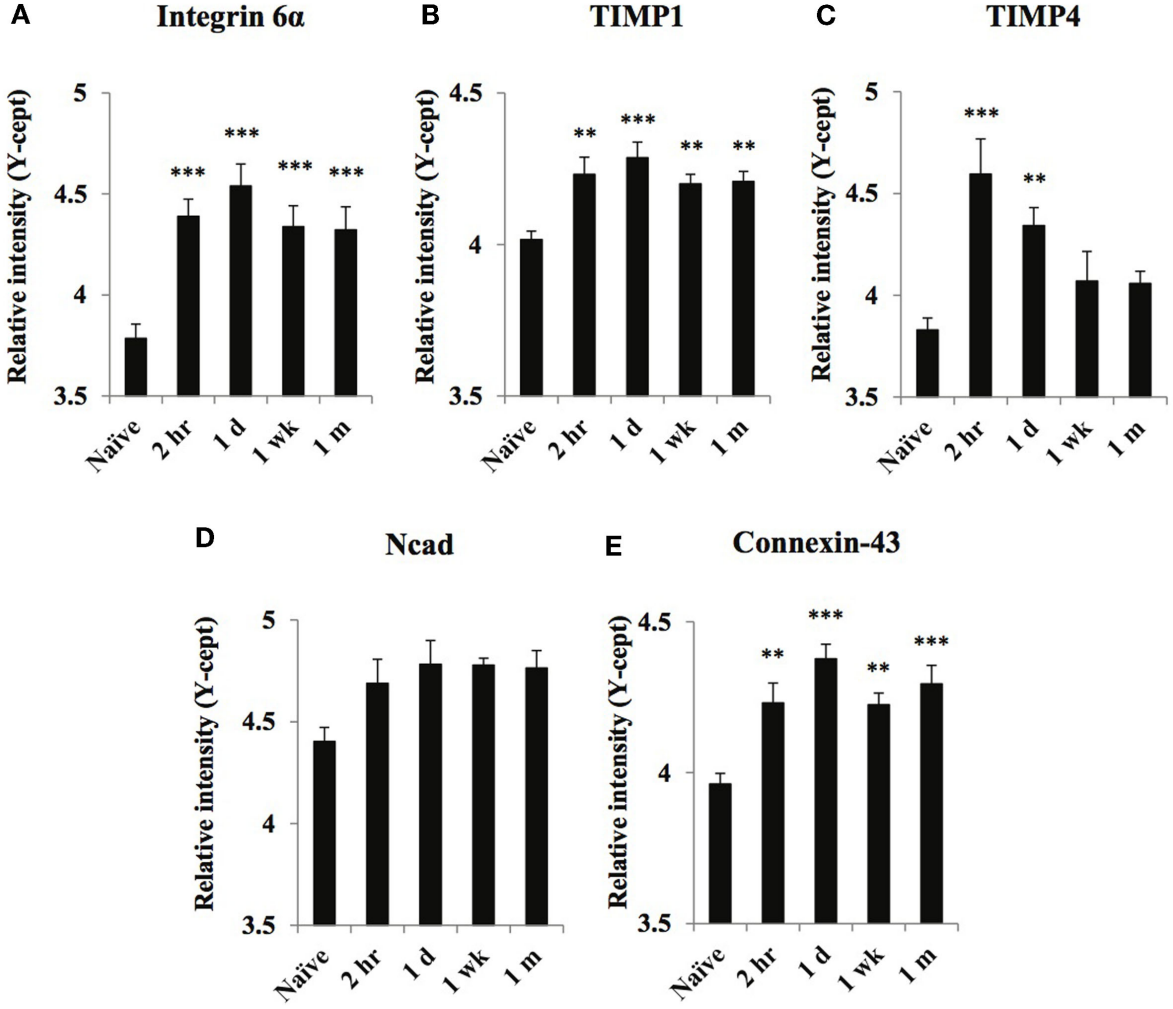
**Temporal changes of select cell surface markers in serum obtained from naïve and at 2 h, 1 day, 1 week, and 1 month of post-blast over pressure exposed animals**. Protein levels of integrin 6α **(A)**, TIMP1 **(B)**, TIMP4 **(C)**, Ncad **(D)**, and Connexin-43 **(E)** were assayed using reverse phase protein microarray (RPPM) and are expressed as *y*-cept values (log 10). Data are presented as the mean ± SEM; **p < 0.01, ***p < 0.001.

**Figure 4:**
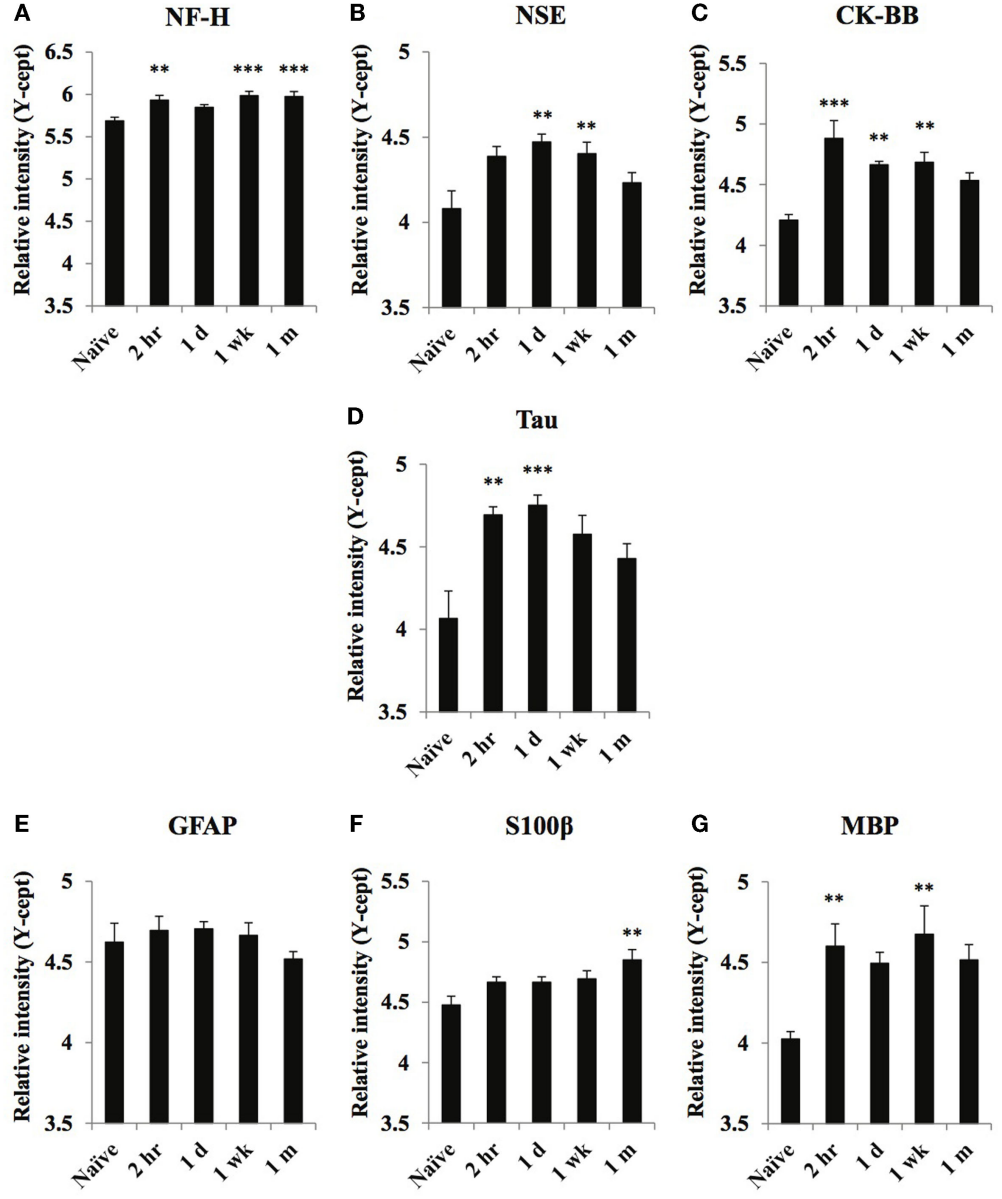
**Temporal changes of select neuronal and glial damage markers in serum obtained from naïve and at 2 h, 1 day, 1 week, and 1 month of post-blast over pressure exposed animals**. Protein levels of neuronal markers NF-H **(A)**, NSE **(B)**, CK-BB **(C)**, Tau **(D)**, glial markers GFAP **(E)**, S100β **(F)**, and MBP **(G)** were assayed using reverse phase protein microarray (RPPM) and are expressed as *y*-cept values (log 10). Data are presented as the mean ± SEM; **p < 0.01, ***p < 0.001.

Differences in protein biomarker levels at each of the post-injury time points relative to naïve levels were analyzed with independent *t-*tests. A Bonferroni-adjusted significance level of 0.0125 was calculated to account for the increased possibility of type I error. Relative intensity data (*y*-cept values) were presented as the mean ± SEM. Statistical significance is reported for naïve vs. injured mice at each time point where applicable (Figures 1–4).

## Results

### Body weight

Blast exposure caused significant weight loss at days 3 and 5 after injury: 27.5 ± 0.65 g and 27.7 ± 0.53 g, respectively, compared to the pre-injury value of 29.3 ± 0.35 g. The body weight (BW) of injured mice was normalized 1 week after the exposure.

### Acute neurological outcome

Following the cessation of anesthesia, control mice recovered the tail pinch reflex after a mean 1.65 ± 0.15 min, corneal reflex after 1.86 ± 0.17 min, and righting reflex after 2.73 ± 0.25 min. The blast exposure somewhat extended the time needed for reflex recovery (tail pinch reflex: 2.43 ± 1.37 min; corneal reflex: 3.37 ± 2.13 min; and righting reflex: 3.42 ± 2.02 min). Nevertheless, these changes were not statistically significant due to relatively large variations between animals.

### Blast injury severity

The mortality rate at 24 h after exposure was 4.5%, which is comparable to the 5% mortality rate in animals exposed to mild intensity blast reported in our previous study (30). Death occurred immediately, mostly due to cardiorespiratory depression and did not correspond to any organ damage. In our current study, the total blast injury severity score was 22.25 ± 3.11 PSS compared to 24.44 ± 0.50 PSS in our original paper (30). Morphological damage in the form of slight petechiae and rare ecchymosis were mainly found in the lungs of exposed mice. Gross pathological observation of the brains following mild blast injury showed an absence of subarachnoid hemorrhage, focal lesions, or contusions.

### General biomarker trends

We found no significant differences in serum biomarker levels between naïve and sham animals. However, the exposure to a single, ­low-intensity blast resulted in substantial changes in the serum levels of a number of biomarkers indicating changes in metabolic and vascular functions, altered cell adhesion, as well as neuronal, axonal, and glial damage. Interestingly, serum levels of the majority of ­inflammatory and astroglial markers did not change, while other biomarkers showed distinct temporal patterns (summarized in Table 1).

**Table 1:** **Summary of injury-induced changes in serum biomarker levels relative to control levels**.

Markers	Post-injury time points			
	2 h	1 day	1 week	1 month
**Metabolism**				
4-HNE	↑	↑	↑	↑
HIF-lα	↑	↑	↑	↑
Ceruloplasmin	↑	↑	↑	↑
**Vascular function**				
AQP1	↑	↑	↑	↑
AQP4	Ø	Ø	Ø	Ø
VEGF	↑	Ø	↑	↑
vWF	↑	↑	↑	↑
Flk-1	↑	↑	Ø	Ø
**Inflammation**				
OPN	↑	↑	Ø	↑
CINC1	Ø	↑	↑	Ø
Fibrinogen	↑	Ø	Ø	Ø
MlP-lα	Ø	↑	Ø	Ø
OX-44	↑	↑	↑	Ø
p38	↑	↑	Ø	Ø
MMP-8	Ø	Ø	Ø	Ø
MCP-1	Ø	Ø	Ø	Ø
CCR5	Ø	Ø	Ø	Ø
CRP	Ø	Ø	Ø	Ø
Galectin-1	Ø	Ø	Ø	Ø
**Cell adhesion and extracellular matrix**				
Integrin α6	↑	↑	↑	↑
TIMP1	↑	↑	↑	↑
TIMP4	↑	↑	Ø	Ø
Ncad	Ø	Ø	Ø	Ø
Connexin-43	↑	↑	↑	↑
**Axonal damage**				
NF-H	↑	Ø	↑	↑
Tau	↑	↑	Ø	Ø
**Neuronal damage**				
NSE	Ø	↑	↑	Ø
CK-BB	↑	↑	↑	Ø
**Glial damage**				
GFAP	Ø	Ø	Ø	Ø
S100β	Ø	Ø	Ø	↑
MBP	↑	Ø	↑	Ø

### Metabolic and vascular markers

Of the three metabolic and oxidative stress markers, ceruloplasmin showed the highest initial increase at 2 h compared to naïve levels; serum levels continued to increase reaching a maximum at 1-month post-injury (Figure 1C). HIF-1α levels were highest at 2 h post-injury, remaining elevated up to 1 month after injury (Figure 1B). 4-HNE serum levels were highest at 1 day and 1-month post-injury (Figure 1A).

Of the markers related to vascular function, serum levels of AQP1, VEGF, and vWF showed the most substantial increases in response to injury, each displaying a distinct temporal pattern (Figures 1D,F,G). The changes in VEGF levels showed a bi-phasic profile. After an initial peak at 2 h, VEGF levels returned close to normal before peaking again at 1-month post-injury (Figure 1F). vWF levels were highest 1 day post-exposure and while serum levels declined over time, they were still significantly higher than naïve values 1 month after the injury. Serum concentrations of the other vascular marker, Flk-1, showed an early increase at 2 h that was somewhat sustained at 1 day; by 1 wk levels returned to normal, control values. We found no significant increase in serum AQP4 levels at any post-injury time points (Figure 1E).

### Inflammatory markers

Serum levels of a few inflammatory markers, OPN, fibrinogen, OX-44, and p38, increased rapidly after injury; marker levels (except OPN) returned to control values at or close to their peak level at 1-month post-injury (Figures 2A,C,E,F). The serum levels of CINC1 and MIP-1α (Figures 2B,D) were increased only at 1 day or 1 week after injury then decreased at 1 month.

Serum levels of MMP-8, MCP-1, CCR5, CRP, and galectin-1 (Figures 2G–K) did not change significantly compared to control values.

### Cell adhesion and extracellular matrix markers

Serum concentrations of all cell adhesion proteins but Ncad increased in response to the injury, although the temporal profiles were different (Figure 3). Serum levels of all markers but TIMP4 reached their maximum at 1-day post-injury and remained elevated until the end of the study. TIMP4 levels were highest at 2 h after injury, decreased by 1 week, and returned to control levels by 1 month (Figure 3C).

### Axonal, neuronal, and glial damage markers

Serum levels of the axon- and neuron-specific markers, NF-H, Tau, NSE, and CK-BB, increased moderately in response to blast injury (Figures 4A–D). At 1 month after injury, NSE, CK-BB, and tau levels returned to control levels, whereas NF-H remained significantly elevated. Serum levels of the astroglia markers GFAP and S100β were not significantly increased at any of the post-injury time points (except for a moderate elevation in S100β at the 1-month time point) (Figures 4E,F). Conversely, MBP levels showed robust increases at 2-h and 1-week post-injury (Figure 4G).

## Discussion

Cognitive and emotional symptoms have been consistently observed in soldiers exposed to blast(s). It has been reported that blast forces alone can cause deleterious behavioral changes when evaluated with selected measures of personality (35). Blast exposure has also been linked to self-perceived cognitive decline in soldiers with mbTBI (36). Recent experimental data support the notion that the exposure to blast (as an emotionally traumatizing event) can alter memory function and cause anxiety (37). Furthermore, it has been recognized that individuals with bTBI also suffer from PTSD (38, 39) and that these two clinical conditions likely develop in parallel leading to a unique and complex clinical entity. Hence, the differential diagnosis between PTSD and bTBI only has merit if it determines distinct therapeutic approaches or if it serves to pre-empt bTBI complications.

The diagnosis of mild blast TBI is hugely misleading; it only indicates the brain’s immediate response to blast and does not take into account the severity of long-term complications. Nevertheless, without better diagnostic nomenclature, we continue to use this term (i.e., mbTBI) both in clinical practice and basic science research. Bearing in mind that there are over 300,000 veterans diagnosed with mbTBI, the timely diagnosis of mbTBI still remains an unsolved problem. Our main goal in this study was to use our previously standardized model and experimental set-up of low-intensity blast exposure generated in laboratory conditions to identify a set of biomarkers that would provide insight into the complex pathobiology of blast-induced neurological deficits (in this paper termed mbTBI to annotate that it was induced by low-intensity primary blast). Chronic elevation in serum levels of vascular and inflammatory biomarkers has been previously found (14, 34), suggesting a profound systemic response to blast exposure; such distinctive biomarker patterns could be useful in differentiating mbTBI from impact TBI and TBI from PTSD.

In our previous study that outlined the main features of our experimental model (30), we compared several shockwave forms: (1) the Friedländer waveform, a simplified qualitative depiction of an explosion-generated shockwave showing positive and negative phases of a pressure wave; (2) an open field shockwave generated by detonating 816.47 g (1.8 lbs) of a TNT-equivalent explosive charge in open field (even terrain, no surrounding objects) conditions. The static pressure was recorded 3.6 m from the source; (3) a complex shockwave form generated by detonating 725.75 g (1.6 lbs) of a TNT-equivalent explosive charge in an urban-environment. The static pressure was recorded 2.3 m from the source; and (4) a moderate intensity shockwave form generated in our shock tube. The static pressure was recorded 4.5 m downstream from the shock tube diaphragm. The blast signatures showed a similarity between the shapes of the shock tube output and the open field shockwave; the intensity of the shock tube-generated overpressure has been modified based on the Bowen curve calculations for small experimental animals.

In this study, we used our previously standardized mouse model of primary blast injuries, generated using a modular, multi-chamber shock tube capable of reproducing complex shock wave signatures seen in theater (30, 31), to elucidate the molecular mechanisms that underlie the neurobehavioral and functional deficits observed clinically and modeled experimentally (20, 28, 40, 41). The experimental set-up in our current study is comparable, albeit not identical to the previous design (30). Nevertheless, the general response to the blast exposure including BW changes, acute neurological recovery, and tissue damage in multiple organs (PSS score) is very similar in both studies. This implies that all other outcome measures (i.e., behavior, motor, and cognitive function) are also comparable in the two studies.

Using antibody-based proteomic analyses, we monitored changes in the serum levels of 31 protein biomarkers up to 1-month post-injury and identified a number of leading pathologies that include metabolic and vascular changes with minimal neuronal and glial damage. More specifically, we collected blood samples from injured mice at 2-h, 1-day, 1-week, and 1-month post-blast to determine the short- and long-term changes in protein markers related to metabolism (4-HNE, HIF-1α, ceruloplasmin), vascular function (AQP1, AQP4, VEGF, vWF, Flk-1), inflammation (OPN, CINC1, fibrinogen, MIP-1α, OX-44, p38, MMP-8, MCP-1 CCR5, CRP, galectin-1), cell adhesion and the extracellular matrix (integrin α6, TIMP1, TIMP4, Ncad, connexin-43), axonal (NF-H, Tau), neuronal (NSE, CK-BB), and glial damage (GFAP, S100β, MBP).

Clinical observations and experimental studies using different TBI models have shown that mTBI is followed by metabolic changes, including oxidative stress (8, 42, 43). In this study, we found that all three metabolic markers responded to mbTBI with rapid and lasting increases. Serum levels of 4-HNE, a byproduct of lipid peroxidation, was significantly increased 2 h after injury and its levels remained elevated at 1-month post-injury. Previous studies of chronic otitis, bipolar disorder, and experimental TBI have found associations between elevated serum 4-HNE levels and oxidative stress (44–47). HIF1α, a transcription factor, is part of organisms’ adaptive response to noxious insults involving brain hypoxia (e.g., TBI and stroke); blocking HIF-1α has been found to ameliorate neuronal and vascular damage (48–51). Ceruloplasmin is involved in facilitating the export of un-bound iron and iron metabolism in the brain (52, 53). Iron metabolism in the CNS must be tightly regulated as excess iron can lead to metabolic stress. TBI causes an elevation of non-heme bound iron independent of hemorrhage or microbleeding; excess iron can trigger the production of reactive oxygen species leading to oxidative stress and inflammation. Therefore, increased ceruloplasmin levels after TBI may reflect a compensatory response to injury-induced increases in free iron (52).

Previous data indicated that the exposure to low-intensity blast can increase serum levels of biomarkers related to various vascular functions (14, 34). The vascular markers we tested in this study, especially VEGF and vWF, showed robust changes of differing temporal patterns in response to blast. VEGF, a trophic factor of endothelial origin, is a key regulator of numerous vascular functions such as the regulation of endothelial permeability, microvascular density, and angiogenesis among others (54–56). Hypoxia is one of the inducers of VEGF expression, and elevated serum levels of VEGF have been associated with various tumors and inflammatory processes. We previously found similarly elevated serum VEGF levels in other animal models of TBI (14, 34). The temporal profile of serum VEGF levels showing a robust and steady increase that peaks at 1-month post-injury suggests continuing vascular changes with a tendency for chronicity. Such a temporal profile may implicate a lasting peripheral/systemic pathological response to low-intensity blast in mice.

The importance of the blast-induced hydrodynamic pulse through venous vasculature has been demonstrated in recently published experimental work by Simard et al. (57). In rats exposed to thorax only blast injury, the authors found perivascular, mainly perivenular, changes in the brain consistent with neuroinflammation: upregulation of TNF-α accompanied by the finding of ED-1-positive cells (macrophages or activated microglia). It has been suggested that the hydrodynamic pulse radiates through vasculature away from its site of origin, ascending easily into the vasculature of the brain via veins since there are no valves to impede pressure transmission. The passage of the pressure throughout the body, besides its direct effects on the vasculature, also activates the autonomous system triggering parasympathetic reflexes causing a sudden drop in blood pressure and heart rate as well as intermittent periods of tachypnea and bradypnea. The ensuing hypoxia caused either by the vagal reflex loop, lung parenchyma damage, or both, further contributes to the changes in systemic and cerebral metabolism as well as in vascular tone.

AQP1 serum levels exhibited a temporal profile similar to that of VEGF. It was significantly increased at 2 h after injury and remained elevated at 1 month. In the CNS, AQP1 is mostly expressed by epithelial cells of the choroid plexus; as the major water-transporting protein in choroid plexus, it is involved in CSF production (58). However, AQP1 is also expressed in several non-neural cells including epithelial cells of the lung and peritoneum among others. (59). Chronically elevated serum levels of AQP1 can indicate injury to peripheral organs, such as the lungs or the GI tract; indeed, their roles have been suggested in the peripheral pathomechanism of bTBI (13, 30).

Serum levels of vWF, another marker of vascular/endothelial stress/damage, also showed a robust response to injury. vWF, an endothelium-specific glycoprotein, is primarily involved in restoring homeostasis in response to endothelial stress, recruiting platelets, and regulating blood coagulation (60, 61). Similar to VEGF, vWF is also involved in the regulation of angiogenesis, cell proliferation, and inflammation (61). Elevated vWF serum level is an indicator of increased vascular permeability in various disease conditions and of unfavorable outcome in TBI (62). Increased vWF serum levels have also been found in systemic inflammation (63, 64).

Consistent with the increase in vWF, we also found significant increases in the serum levels of several inflammatory markers in response to low-intensity blast. The levels of OPN remained elevated at 1 month after injury indicating that low-intensity blast might trigger a chronic inflammatory process. This finding is consistent with our previous observation in the same model of bTBI, which demonstrated a substantial ongoing systemic inflammatory process that included the CNS (30). Elevated OPN levels can indicate an autoimmune process as seen in multiple sclerosis (65–67).

CINC1 is produced by astrocytes in response to various noxious stimuli, including oxidative stress (68). Elevated serum levels of fibrinogen, a plasma glycoprotein, are another indication of altered microcirculation and inflammation; increased levels of fibrinogen can increase blood viscosity as well as vascular permeability (69, 70). According to studies showing correlation between elevated fibrinogen precursor protein and Alzheimer’s disease, elevated fibrinogen levels can link inflammation and vascular damage to neurodegeneration. MIP1α is a proinflammatory chemokine, and its elevated serum levels are yet another indicator of inflammation (71).

Inflammation can also be triggered by injury-induced changes in cellular adhesion (72, 73). The transient increase in serum levels of cell adhesion/cell surface markers can be an indication of low-intensity blast’s ability to interrupt cellular connectivity, which may in turn contribute to the initiation and/or potentiation of the inflammatory response. The blast-induced cell surface disruption seems to be transient as shown by the early peaks in integrin α6, TIMP1, TIMP4, Ncad, and connexin-43 serum levels. At 1 month after injury, levels of TIMP4 and Ncad were back or close to control values. TIMP4 is an inhibitor of matrix metalloproteinases involved in the degradation of the extracellular matrix, thus, cell surface remodeling can be part of the response to low-intensity blast exposure (74–76). N-cadherin, a multifunctional molecule with important roles in cell adhesion, is also involved in mediating astrogliosis (77, 78).

The changes in serum protein biomarkers discussed above can reflect a systemic or at least partly systemic molecular response to blast. Indeed, previous studies have shown that the exposure to blast triggers complex systemic responses, including a systemic inflammatory response and altered physiological functions (13, 14, 30). These – and other systemic changes – can contribute significantly to the pathomechanisms of mbTBI. In addition to the systemic response to blast, we found indications of axonal, neuronal, and glial damage. Our previous works have demonstrated that the exposure to a single low-intensity blast can trigger changes in the serum levels of markers indicating neuronal and astroglial damage (17, 20, 23, 79, 80). A recent clinical study found elevated serum levels of ubiquitin C-terminal hydrolase-L1 (UCH-L1), αII-spectrin breakdown products (SBDPs), and GFAP in soldiers repeatedly exposed to low-intensity explosive blast indicating damage to neuronal and glial structural integrity (81).

Serum levels of the neuronal markers NSE and CK-BB were only increased during the acute and subacute post-injury period, and returned to normal levels at 1 month. Of the two axonal damage markers, the serum levels of NF-H showed rapid and sustained increases, whereas Tau levels were increased only during the acute phase of injury (2 h and 1 day). Elevated serum levels of various neurofilament proteins have been found to clinically correlate with injury severity; as such, they could be useful in predicting injury outcome (82). In a large animal model of bTBI, rapid rise of serum NF-H levels has been shown to correlate with unfavorable outcome (79). Clinically, elevated serum levels of total tau have been found to be good and bad predictors of outcome after mTBI (83–86). It should be noted that clinical studies typically analyze serum levels of protein biomarkers (e.g., tau) at a single – and not always at the same – post-injury time point(s). As our current study illustrates, serum levels of protein biomarkers change as a function of time elapsed after injury. Therefore, a negative finding based on a single measurement has little diagnostic (or scientific) value. An elegant experimental study has shown that tissue levels of various tau proteins, including cleaved and phosphorylated forms, were increased at the 24-h post-injury time point and remained increased at 30 days in the brains of mice exposed to mild blast (87). These finding implicate tau pathology as one of the long-term consequences of a single mild bTBI, but in the absence of serum data it is unclear how intracerebral findings translate into changes detectable in the serum. Furthermore, dysfunction of the glymphatic pathway (triggered by TBI) can substantially affect the intracerebral accumulation of tau and modify its serum levels as discussed later (88).

The sustained increase in serum NF-H levels, especially in the light of the similarly elevated MBP levels, indicates ongoing white matter damage. White matter injury detected by diffusion-tensor imaging (DTI) has been identified as a hallmark of mTBI (21, 89, 90). Indeed, in our current study as well as previous experiments, we have found indications of myelin damage reflected by elevated serum MBP levels (79). Both experimental and clinical imaging studies have found white matter tract damages following blast exposure(s) (89, 91, 92). The exact pathology that can cause and or sustain long-term axonal damage is currently not fully understood, but inflammatory responses have been implicated in the long-term effect of bTBI (93).

Of the astroglial markers, only S100β levels increased significantly and only at the 1-month time point. Astrogliosis, one of the hallmarks of TBI, represents a neuroprotective response to injury (94). Intriguingly, GFAP levels did not increase significantly at any of the measured time points. These somewhat conflicting findings can be explained by the fact that S100β is a cytoplasmic and partially membrane-bound protein, whereas GFAP is a cytoskeletal protein. Although the exact relationship between physical forces and structural damage at the molecular level is not known, one can hypothesize that cytoplasmic proteins can likely be released more easily from mildly damaged cells upon physical impact than structural proteins such as GFAP. Cytoskeletal proteins may require larger physical/kinetic forces to damage the cytoskeletal network as well as the cellular membrane to enable the release of GFAP into the extracellular space. It should be noted that S100β is not entirely astroglia-specific, so the detected increases can be from other non-neural cells (95, 96). Nevertheless, clinical observations have shown that increased serum S100β levels of non-neural origin are typically observed at the earliest post-injury time points, whereas the delayed increases in serum S100β are usually of neural origin and have been associated with unfavorable outcome in severe TBI (97). In contrast to GFAP, MBP levels increased substantially at all post-injury time points, and remained close to its peak level at 1 month after injury. Thus, our findings implicate lasting myelin as well as axonal damage in the pathobiology of mbTBI as discussed above. These findings are consistent with previous clinical imaging studies showing chronic white matter damage in military personnel exposed to blast (90, 92).

It has been assumed that the elevated serum levels of neural-specific protein biomarkers also reflect damage and/or at least a temporary opening of the blood–brain barrier (BBB) in TBI. BBB opening, however, is associated with moderate or severe TBI, and mild TBI typically does not increase BBB permeability. This paradox has been solved with the recent discovery of the brain glymphatic system (98). In this model, the CSF moving through the glymphatic pathway transports biomarkers to the blood via the cervical lymphatic system, subsequently ending up in the systemic circulation. According to this model, no BBB disruption is needed for proteins released from damaged neural cells to reach the systemic circulation. However, the brain-to-blood transport of protein biomarkers depends on the activity of glymphatic system and this activity is regulated by multiple clinically relevant factors such as sleep deprivation (99). Future experiments analyzing injury-induced changes in both the serum and the CSF can provide critical information about the relationship between intra- and extra-cranial pathologies.

## Conclusion

The extremely complex nature of blast injuries requires full understanding of blast physics and effects, and a model reproducing multiple aspects of blast injuries should be defined with particular scientific fidelity to conditions observed in theater. Otherwise, a model will lack military and clinical relevance and the obtained results might be dangerously misleading (11, 100). Thus, we strongly believe that if we aspire to understand the origin of blast-induced acute and chronic neurological deficits, we should reproduce the injurious environment our soldiers are exposed to as close as possible: this also requires reproducing the main response mechanisms to blast exposure(s) – systemic, local, and cerebral – as reported in clinical studies.

There is a continuing discussion about adequate scaling laws that would reliably estimate the exposure conditions causing comparable injuries in humans and animals. Many of the new suggestions about scaling laws focus on the physical mechanisms that determine brain response to blast or differences in properties of soft tissues of the head or the major anatomical differences, particularly of the skull (101–103). Nevertheless, these approaches fail to take into account the specificity of the animals’ physiological tolerance toward blast exposure, which often differs from the human tolerance. Indeed, the human body can survive relatively high blast overpressure without experiencing barotrauma. A 5-psi blast overpressure would rupture eardrums in about 1% of subjects, whereas the threshold for lung damage occurs at about 15-psi blast overpressure and a 35–45 psi overpressure may cause 1% fatalities (104). Hence, in our model, we used the Bowen animal-to-human scaling laws for blast effects on the animals, which is based on mass scaling, to set the basic parameters for shockwave exposure; then, we fine tuned to conditions to reach the clinical features of blast injuries and blast-induced traumatic brain injuries described in humans exposed to blast (6, 24, 25, 105–107).

Although the research community’s interest is almost entirely focused on neurological deficits due to blast, which grouped together often called bTBI and a “signature wound” of recent wars, accumulating evidence provides more and more support to the importance of systemic, multi-organ mechanisms in the pathobiology of blast-induced neurological deficits. Bearing this in mind, we used the previously developed, well-standardized, and validated mouse model of primary blast injuries (30), which reproduces the main consequences of blast exposures: changes in general physiology, multi-organ damages, systemic compensatory mechanisms, as well as neurological deficits seen in individuals exposed to differing blast intensity levels. Thus, our intention was never to reproduce only a head injury (i.e., isolated TBI) in classical terms, but the full spectrum of injury response mechanisms induced by primary blast. These include the autonomous system activation including the parasympathic reflexes; hypoxia caused either by the vagal reflex loop, lung parenchyma damage or both; changes in vascular tonus or systemic and/or cerebral metabolism – all these, rather than direct brain parenchyma damage, might underlie the blast-induced neurological deficits. Indeed, our current data provide further support to the importance of systemic changes in the pathobiology of blast-induced neurological deficits.

In summary, this is the first study to determine the temporal pattern of changes in the serum levels of protein biomarkers after a single exposure to low-intensity blast in the high-fidelity mouse model of bTBI. Our findings implicate oxidative stress, vascular changes, inflammation, altered cell adhesion, neuronal, and glial damage/loss in the pathobiology of bTBI. The observed temporal profiles of markers illustrate the dynamic nature of numerous molecular mechanisms related to the systemic and cerebral response to blast. Our work is limited by the incomplete understanding of the physics of explosive blast, how the physical event translates into biological response(s) and importantly, how well rodent models replicate the human condition. Proteolytic cleavage of protein biomarkers is also a potential issue for Reverse Phase Protein Microarray (RPPM) like all other antibody-based systems, such as the ELISA, Luminex, or Western blots. Nonetheless, our findings underline the importance of monitoring serum biomarker levels over a prolonged period of time. Determining the temporal pattern of molecular changes can provide critical information about the progression or regression of the individual pathomechanisms and help in the development of targeted and timely therapeutic interventions for improved prognosis.

## Author Contribution

FA performed the animal experiments, collected and processed the biosamples, performed the proteomic analysis of the samples, analyzed the data, generated the figures, and wrote the first draft. IC designed and supervised the blast experiments, co-wrote, and edited the manuscript. SP performed part of the animal experiments, assisted FA with collecting the biosamples, and reviewed the manuscript. DA designed the proteomics analysis, analyzed and interpreted the data, and wrote the manuscript.

## Conflict of Interest Statement

No conflicting financial interests exist. The views, opinions, and/or findings contained in this article are those of the authors and should not be interpreted as representing the official views or policies, either expressed or implied, of the Department of Defense (DoD). Approved for public release, distribution unlimited.

## Supplementary Material

The Supplementary Material for this article can be found online at http://journal.frontiersin.org/article/10.3389/fneur.2015.00114/abstract
