# Supplementary material for: The Temporal Pattern of Changes in Serum Biomarker Levels Reveals Complex and Dynamically Changing Pathologies after Exposure to a Single Low-Intensity Blast in Mice
Source: Front Neurol. 2015 Jun 12;6:114. doi: 10.3389/fneur.2015.00114 (PMC4464198; doi:10.3389/fneur.2015.00114)
Supplement: Supplementary file 1 [file Table_1.PDF]

SUPPLEMENTARY TABLE

Markers	Catalog Number	Company Name	Dilution
**Metabolism**			
4-Hydroxy-2 noneal (4-HNE)	393207	Calbiochem	1:100
Hypoxia-inducible factor-1α (HIF-1α)	SC-53546	Santa Cruz	1:20
Ceruloplasmin	GTX28813*	GeneTex	1:20
**Vascular Function**			
Aquaporin 1 (AQP1)	ab87845	Abcam	1:50
Aquaporin 4 (AQP4)	ab46182	Abcam	1:50
Vascular endothelial growth factor (VEGF)	ab53465	Abcam	1:50
von Willebrand factor (vWF)	SC-8068	Santa Cruz	1:20
FLK-1/VEGF Receptor2	SC-315	Santa Cruz	1:20
**Inflammation**			
Osteopontin (OPN)	SC-73631	Santa Cruz	1:20
Cytokine-induced neutrophil chemoattractant 1 (CINC1)	GTX10365	GeneTex	1:15
Fibrinogen	SC-69775	Santa Cruz	1:20
Macrophage inflammatory protein-1α (MIP-1α)	SC-166911	Santa Cruz	1:20
OX-44/CD53	GTX76102	GeneTex	1:20
p38	9212	Cell Signaling	1:100
Matrix metalloproteinase-8 (MMP-8)	SC-50384	Santa Cruz	1:20
Monocyte chemotactic protein-1 (MCP-1)	SC-1784	Santa Cruz	1:20
C-C chemokine receptor type 5 (CCR5)	GTX61751	GeneTex	1:20
C-reactive protein (CRP)	SC-30047	Santa Cruz	1:20
Galectin-1	GTX62666	GeneTex	1:20
**Cell Adhesion and Extracellular Matrix**			
Integrin α6	GTX100565	GeneTex	1:100
Tissue inhibitor of metalloproteinase 1 (TIMP1)	GTX108254	GeneTex	1:100
Tissue inhibitor of metalloproteinase 1 (TIMP4)	GTX114942	GeneTex	1:100
Ncadherin (Ncad)	SC-31031	Santa Cruz	1:20
Connexin-43	ab63851	Abcam	1:50
**Axonal Damage**			
Neurofilament-H (NF-H)	N4142	Sigma	1:20
Tau	SC-1995	Santa Cruz	1:20
**Neuronal Damage**			
Neuron specific enolase (NSE)	ab53025	Abcam	1:50
Creatine kinase-B type (CK-BB)	SC-15157	Santa Cruz	1:20
**Glial Damage**			
Glial fibrillary acidic protein (GFAP)	ab7260	Abcam	1:500
S100β	SC-13914	Santa Cruz	1:20
Myelin basic protein (MBP)	ab41548	Abcam	1:20
